# Social isolation prevalence estimates and trends over time: it depends on what you measure

**DOI:** 10.1093/geroni/igaf122.1629

**Published:** 2025-12-31

**Authors:** Yulin Yang, Karla Renata, Flores Romero, Irena Cenzer, Carla Perissinotto, Mary Thoma, Ruijia Chen, Jacqueline Torres, Ashwin Kotwal

**Affiliations:** University of California, San Francisco, San Francisco, California, United States; University of California, San Francisco, San Francisco, California, United States; University of California, San Francisco, San Francisco, California, United States; University of California, San Francisco, San Francisco, California, United States; University of California, San Francisco, San Francisco, California, United States; Boston University School of Public Health, Boston, Massachusetts, United States; University of California, San Francisco, San Francisco, California, United States; University of California, San Francisco, San Francisco, California, United States

## Abstract

Social isolation is common among older adults and worsened during COVID-19, but its population-level trends surrounding the pandemic remain unclear. Variations in the measures of social isolation across surveys may contribute to discrepancies in prevalence estimates over time. This study examines social isolation prevalence using two population-based studies, comparing pre-pandemic and pandemic periods and evaluating variation across population subgroups. We analyzed data from the Health and Retirement Study (HRS, 2016–2022) and the National Health and Aging Trends Study (NHATS, 2019–2022). Social isolation was measured across three domains: household and core contacts, social network interaction, and community engagement. We estimated survey-weighted prevalence, adjusting for socio-demographic covariates, and evaluated subgroup differences by socio-demographics (e.g., race and ethnicity). In the HRS, social isolation prevalence increased from 12% in pre-pandemic years (2016, 2018) to 12.9% in 2020 and 13.5% in 2022. In the NHATS, prevalence rose from 4.1% in 2019 to 9.2% in 2020, then declined to 5.6% in 2021–2022. Subgroup differences in social isolation varied by study and time period. In the HRS, non-Hispanic white participants had the highest prevalence compared to non-Hispanic Black and Hispanic participants both before and during the pandemic. In contrast, Black and Hispanic participants had higher prevalence in the NHATS, with disparities shifting over time. Social isolation is a pressing public health challenge for older adults, yet its prevalence varies widely across studies due to measurement differences. Measurement differences should be reconciled to accurately track trends, identify at-risk populations, and inform targeted interventions.

